# Publisher Correction: Elementary steps in electrical doping of organic semiconductors

**DOI:** 10.1038/s41467-018-04275-9

**Published:** 2018-06-12

**Authors:** Max L. Tietze, Johannes Benduhn, Paul Pahner, Bernhard Nell, Martin Schwarze, Hans Kleemann, Markus Krammer, Karin Zojer, Koen Vandewal, Karl Leo

**Affiliations:** 1 Dresden Integrated Center for Applied Physics and Photonic Materials, Technische Universität Dresden, Nöthnitzer Strasse 61, 01187 Dresden, Germany; 2 Physical Science and Engineering Division, KAUST Solar Center, King Abdullah University of Science and Technology, Thuwal, 23955-6900 Saudi Arabia; 3 NAWI Graz, Institute of Solid State Physics, Graz University of Technology, Petersgasse 16, 8010 Graz, Austria; 4 Present Address: Department of Microbial and Molecular Systems, Centre for Surface Chemistry and Catalysis, KU Leuven—University of Leuven, Celestijnenlaan 200F, B-3001 Leuven, Belgium; 5 Present Address: Instituut voor Materiaalonderzoek, Hasselt University, Wetenschapspark 1, 3590 Diepenbeek, Belgium

Correction to: *Nature Communications* 10.1038/s41467-018-03302-z, published online 21 March 2018

The original version of this Article contained an error in Eq. 1. A factor of ‘*c*’ was included in the right-hand term. This has been corrected in the PDF and HTML versions of the Article.

$$p = N_A^ - = c\frac{{N_A}}{{1 + {\mathrm{exp}}\left( {\frac{{E_A - E_F}}{{k_BT}}} \right)}}$$

The correct form of Eq. 1 is:

$$p = N_A^ - = \frac{{N_A}}{{1 + {\mathrm{exp}}\left( {\frac{{E_A - E_F}}{{k_BT}}} \right)}}$$

This has been corrected in the PDF and HTML versions of the Article.

