# Supplementary figures and images for: Elementary steps in electrical doping of organic semiconductors
Source: Nat Commun. 2018 Mar 21;9:1182. doi: 10.1038/s41467-018-03302-z (PMC5862893; doi:10.1038/s41467-018-03302-z)

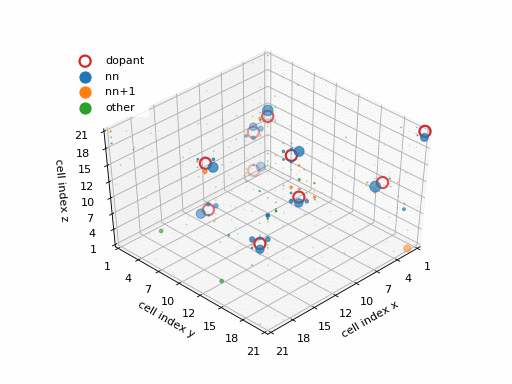

Supplement: Supplementary file 1 — Supplementary Movie 1 [file 41467_2018_3302_MOESM1_ESM.gif]
