# Supplementary material for: Elementary steps in electrical doping of organic semiconductors
Source: Nat Commun. 2018 Mar 21;9:1182. doi: 10.1038/s41467-018-03302-z (PMC5862893; doi:10.1038/s41467-018-03302-z)
Supplement: Supplementary file 2 — Description of Additional Supplementary File [file 41467_2018_3302_MOESM2_ESM.pdf]

Description of Additional Supplementary File

File Name: Supplementary Movie 1

Description: **Monte Carlo transport simulations on ZnPc:F_6-TCNNQ:** Illustration of the simulated occupation probability *W* of sites with charge carriers (holes) in a 21x21x21 mesh with periodic boundary conditions and $MR=10^{-3}$ (10 dopants, without traps). Blue and orange dots represent the occupation probabilities of nearest neighbor (nn) and next nearest-neighbor (nn+1) sites of dopants, respectively, which positions are marked as red circles. For all other sites, the occupation probability is indicated by green dots. The occupation probability *W* increases with the plotted dot size each, here shown for a threshold of $W>10^{-4}$.
