# Supplementary material for: Elementary steps in electrical doping of organic semiconductors
Source: Nat Commun. 2018 Mar 21;9:1182. doi: 10.1038/s41467-018-03302-z (PMC5862893; doi:10.1038/s41467-018-03302-z)
Supplement: Supplementary file 4 — Supplementary Information [file 41467_2018_3302_MOESM4_ESM.pdf]

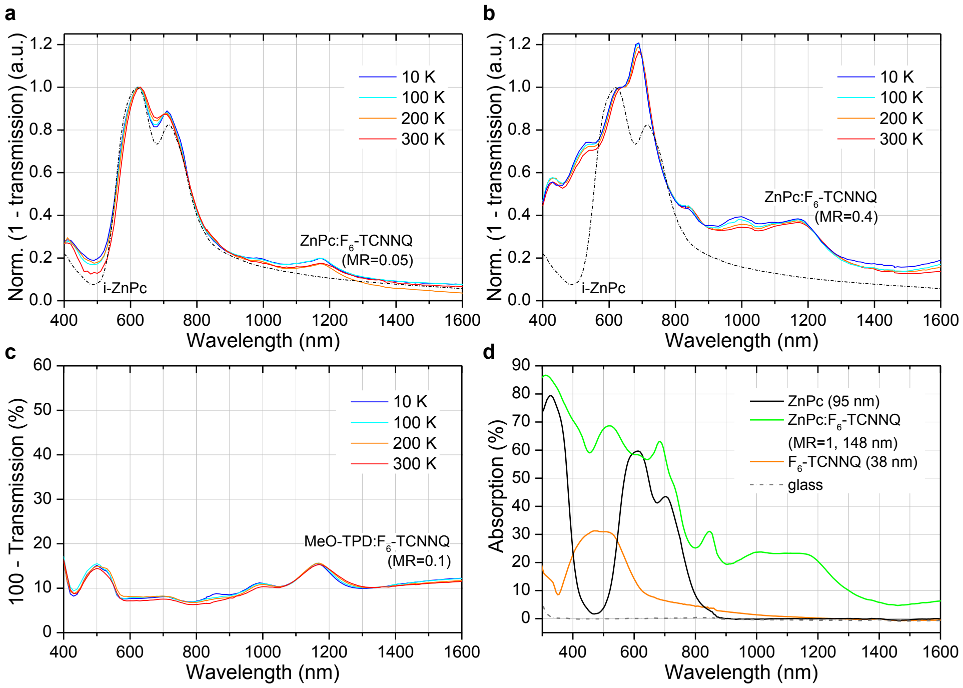

Supplementary Figure 1. Temperature-dependent transmission on p-doped films. Transmission spectra of **(a,b)** ZnPc:F_6-TCNNQ and **(c)** MeO-TPD:F_6-TCNNQ thin films with different doping ratios measured under temperature variation in-between $T = 10\,\mathrm{K} \ldots 300\,\mathrm{K}$ (solid lines). Reference spectra of undoped ZnPc film are shown as dash-dotted lines. **(d)** RT absorption spectra of pure ZnPc and F_6-TCNNQ films as well as of an highly doped ZnPc:F_6-TCNNQ ($MR = 1$) sample.

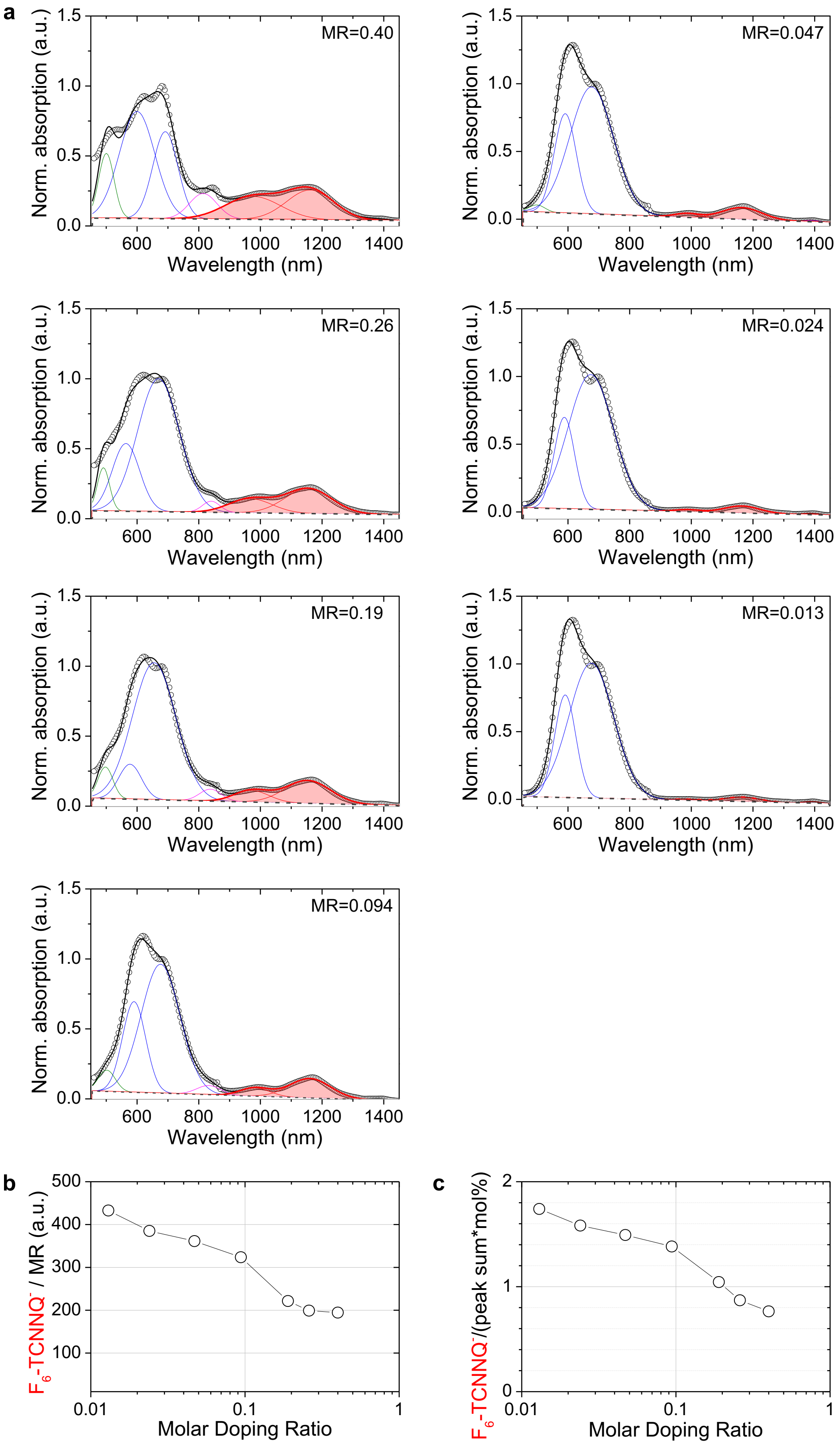

Supplementary Figure 2. Fitting absorption spectra of p-doped films. (a) Gaussians peak fits of the normalized RT absorption spectra of ZnPc:F_6-TCNNQ thin films of varying doping ratio. Red peaks at 995 and 1160 nm are sub-gap features attributed to ionized F_6-TCNNQ$^-$ molecules. **(b)** Integrated fitted intensity of the red F_6-TCNNQ$^-$ peaks weighted by the molar doping ratio MR $= N_A/N_V$. **(c)** Determined ratio $Abs(\mathrm{F_6\text{-}TCNNQ^-})/(Abs(\text{peak sum})\times c[\text{mol\%}])$ using the doping concentration $c[\text{mol\%}] = N_A/(N_A + N_V)$.

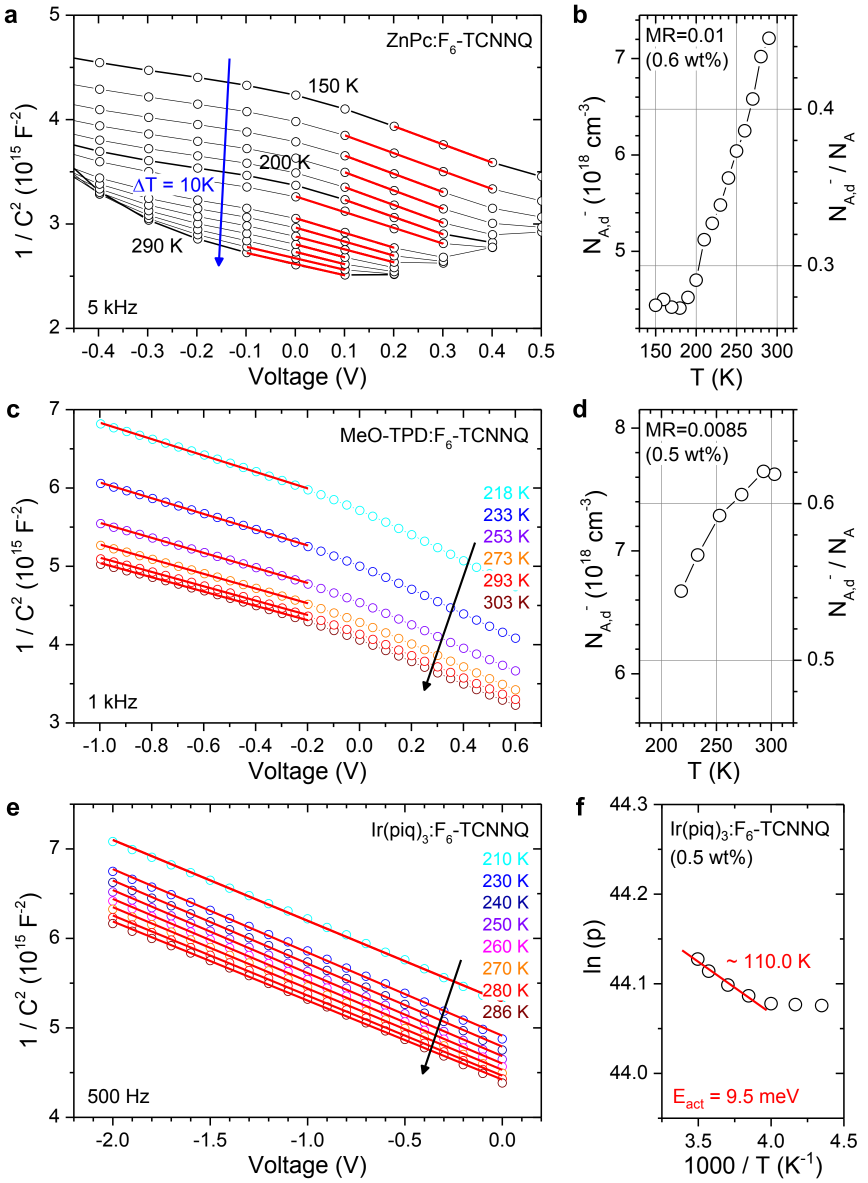

Supplementary Figure 3. Mott-Schottky analysis. $1/C^2(V)$ spectra of ITO/host:F$_6$-TCNNQ(50 nm)/Al Schottky diodes determined by Impedance spectroscopy under varying temperature employing either **(a)** ZnPc or **(c)** MeO-TPD as host material. Red lines indicate linear fits for determining the density of ionized acceptors $N_{A,d}^-(T)$, forming the space charge layer at the metal/organic contacts due to depletion of free carriers p. Molecule densities $N_0 = 1.62 \times 10^{21}\,\mathrm{cm}^{-3}$ $(1.45 \times 10^{21}\,\mathrm{cm}^{-3})$ for ZnPc (MeO-TPD) are used in the molar doping ratio MR $= N_A/N_0$. Respective determined doping efficiencies $N_{A,d}^-/N_A$ are shown in **(b,d)**. Furthermore, Mott-Schottky plots determined on an ITO/Ir(piq)$_3$:F$_6$-TCNNQ(50 nm, 0.5 wt%)/Ir(piq)$_3$(8 nm)/Al device and the respective thermal activation are shown in **(e,f)**. The ionization energy of the amorphous Ir(piq)$_3$ measured by UPS is 5.03 eV [1], i.e., slightly lower than that of MeO-TPD ($IE = 5.07$ eV).
The uncertainty of the Arrhenius-type activation energy E_{act} determined by fitting Mott-Schottky plots is estimated to 5 meV by statistics on 3 ZnPc:F$_6$-TCNNQ diodes.

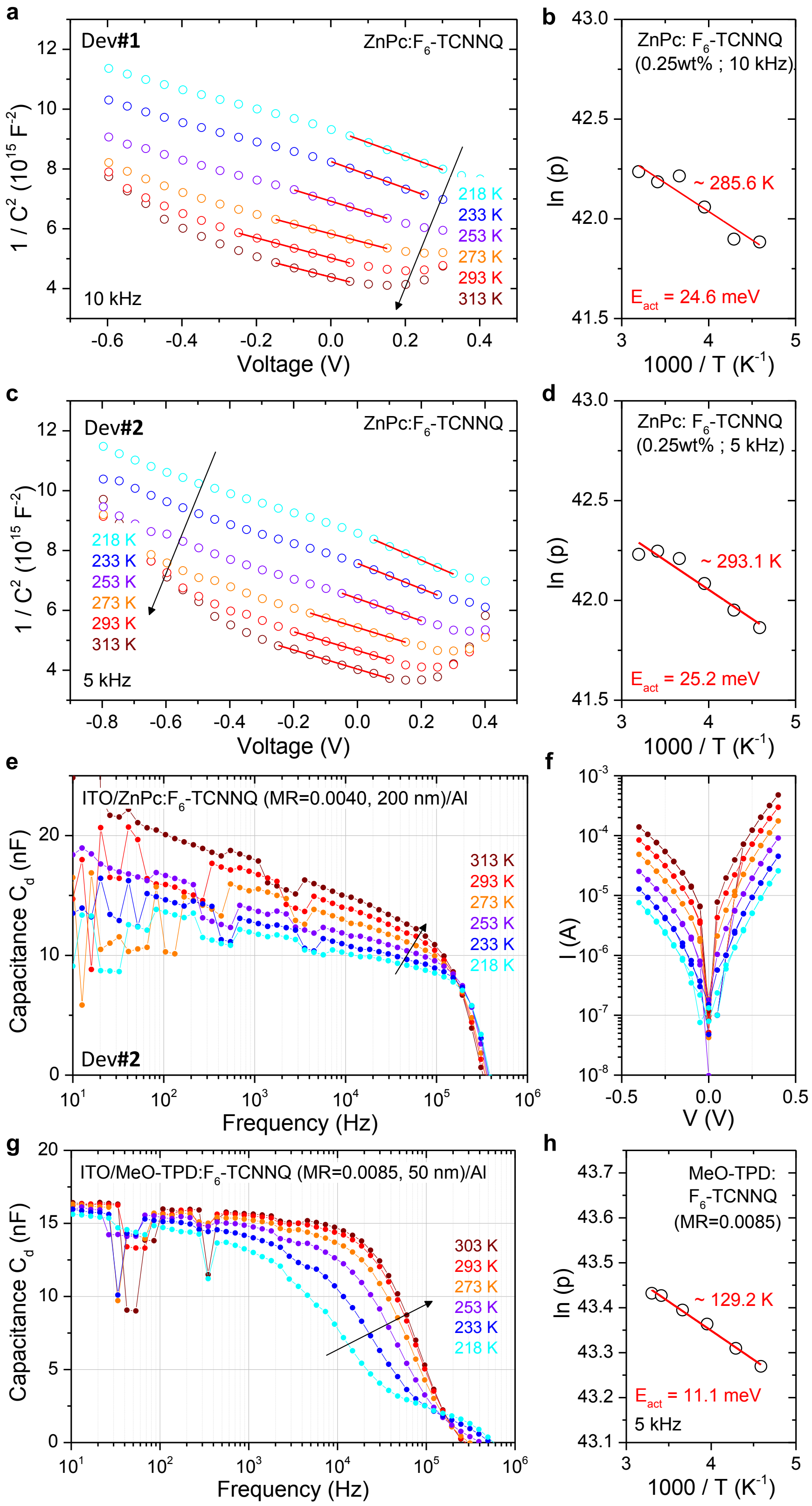

Supplementary Figure 4. Impedance spectroscopy. $1/C^2(V)$ spectra **(a,c)** and Arrhenius-type doping activation **(b,d)** of ITO/ZnPc:F_6-TCNNQ(200 nm, 0.5 wt%)/Al Schottky diodes. Respective $C(f)$ spectra at zero bias and $I(V)$ curves are shown in **(e)** and **(f)**, respectively. **(g)** $C(f)$ spectra of an MeO-TPD:F_6-TCNNQ Schottky diode at zero bias and varying sample temperature for comparison. **(h)** Mott-Schottky analysis at 5 kHz yields an Arrhenius-type activation of 11.1 meV.

Supplementary Note 1. Two- & Three-level System: Fermi level & Doping Efficiency

For given dopant (acceptor) density N_A and temperature T, the generalized neutrality condition

$$p(T) + N_{\mathrm{CT}}^{+}(T) = N_A^{-}(N_A, T) \tag{1}$$

is presumed, in which N_{CT}^{+} denotes the number of holes bound in [$\mathrm{D^+A^-}$] ground-state integer-charge transfer complexes (ICTCs) with binding energy E_{CT}^{b} and density N_A^{-}:

$$N_{\mathrm{CT}}^{+}(T) = N_A^{-}(N_A, T)\left(1 - \frac{1}{1+\exp\left(\frac{E_{\mathrm{CT}}^{b}-E_F}{k_B T}\right)}\right) \tag{2}$$

Thus, we get

$$\begin{aligned} p(T) =& N_A^{-}(N_A, T) - N_A^{-}(N_A, T)\left(1 - \frac{1}{1+\exp\left(\frac{E_{\mathrm{CT}}^{b}-E_F}{k_B T}\right)}\right) \\ =& \frac{N_A^{-}(N_A, T)}{1+\exp\left(\frac{E_{\mathrm{CT}}^{b}-E_F}{k_B T}\right)} \equiv N_{\mathrm{CT}}^{-}(N_A^{-}, T)\,. \end{aligned} \tag{3}$$

The expression on the right hand side corresponds to an "occupation" of [$\mathrm{D^+A^-}$] ICTCs with electrons, i.e., to the density of those being actually separated or isolated ionized dopant molecules. Therefore, it is denoted as N_{CT}^{-}, and for all ionized dopants the density condition

$$N_{\mathrm{CT}}^{-} + N_{\mathrm{CT}}^{+} = N_A^{-} \tag{4}$$

follows.

Ideally, all dopants are ionized: $N_A^{-} = N_A$. Exemplary solutions are shown in Supplementary Figure 6 and 8. For the case of weak dopants, however, incomplete dopant ionization is considered by applying

$$N_A^{-}(N_A, T) = \frac{N_A}{1+\exp\left(\frac{E_A-E_F}{k_B T}\right)} \tag{5}$$

in Supplementary Equation (1)-(4). Respective solutions are shown in Supplementary Figure 9 for varying $E_A = 0.1 \ldots 0.5\,\mathrm{eV}$ and fixed binding energy $E_{\mathrm{CT}}^{b} = 0.64\,\mathrm{eV}$ of holes in ICTCs.

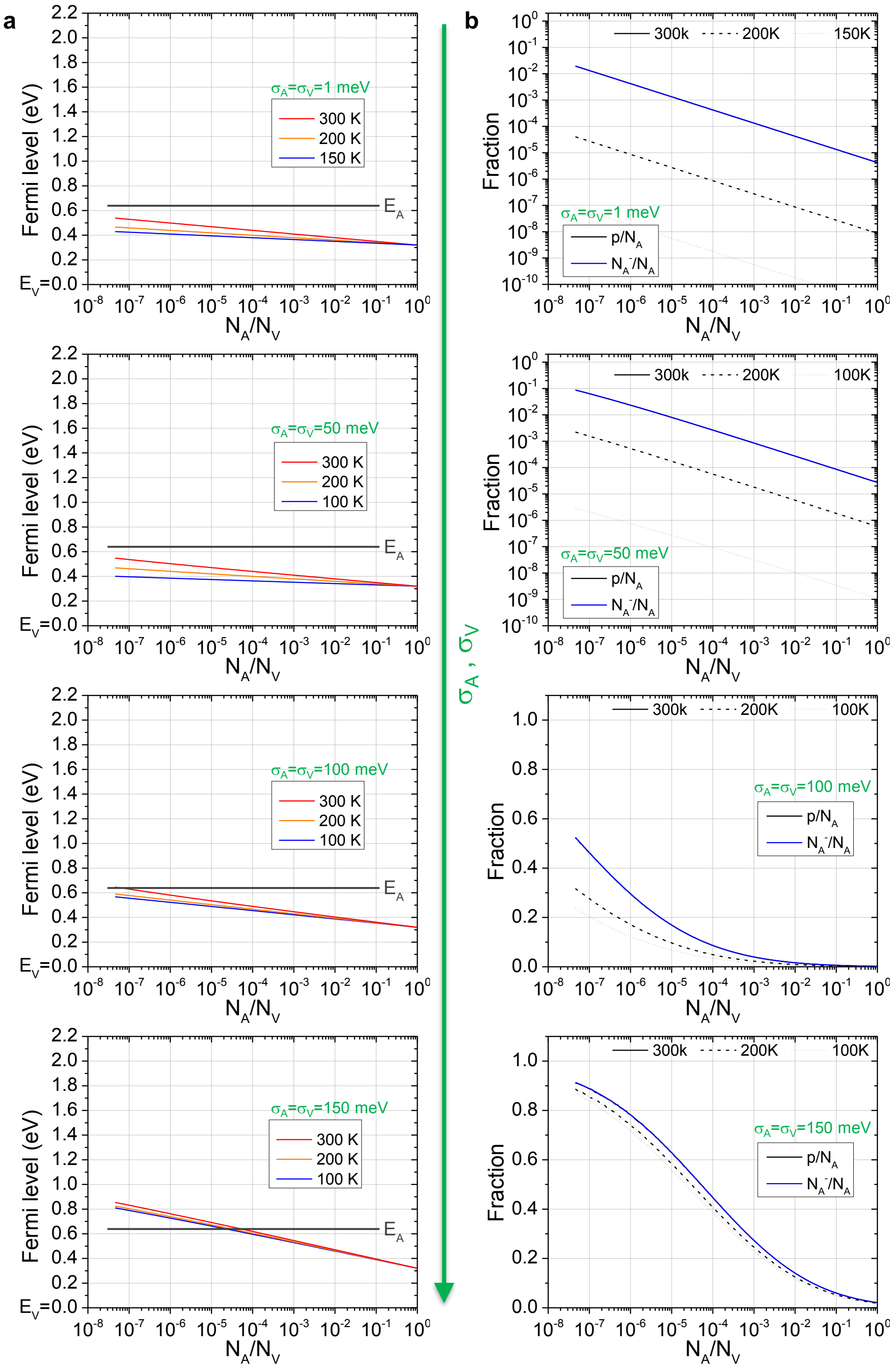

Supplementary Figure 5. Doping model with activation from acceptor states E_A. **(a)** Fermi level and **(b)** doping efficiency *vs.* the acceptor density N_A for a two level system consisting of two broadened Gaussians separated by $E_A - E_V = 0.64\,\mathrm{eV}$. The neutrality condition $p = N_A^-$ is solved. The effective valence density is $N_V = 2.4 \times 10^{19}\,\mathrm{cm}^{-3}$. Temperature T and the widths $\sigma_A = \sigma_V = 1 \ldots 150\,\mathrm{meV}$ are varied.

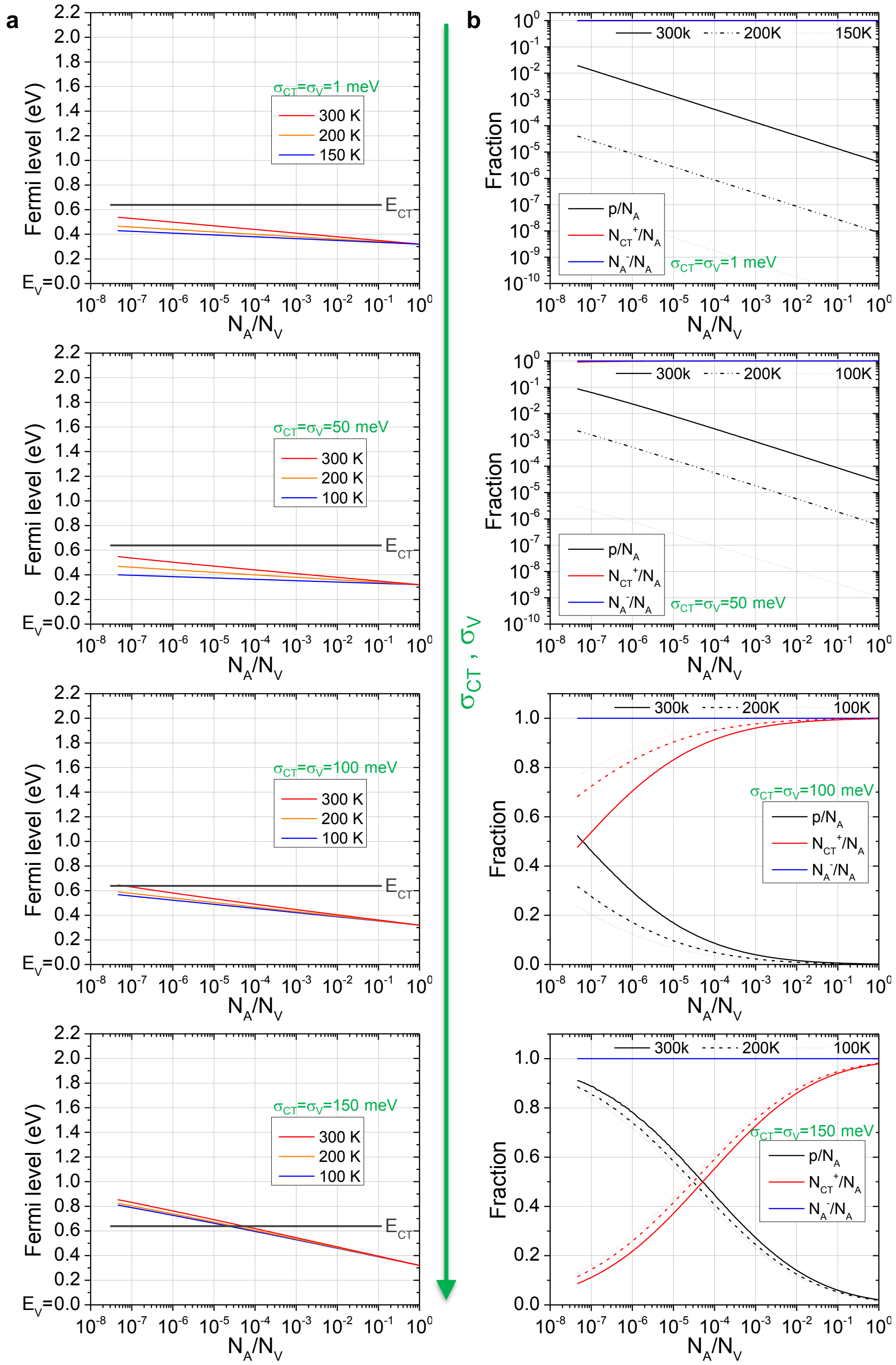

Supplementary Figure 6. Doping model with activation from ICTCs with E^b_{CT}. **(a)** Fermi level and **(b)** doping efficiency *vs.* the acceptor density N_A for a two level system consisting of two broadened Gaussians separated by $E^b_{\mathrm{CT}} - E_V = 0.64\,\mathrm{eV}$. The neutrality condition $p + N^+_{\mathrm{CT}} = N^-_A = N_A$ is solved. The effective valence density is $N_V = 2.4 \times 10^{19}\,\mathrm{cm}^{-3}$. Temperature T and the widths $\sigma_{\mathrm{CT}} = \sigma_V = 1 \ldots 150\,\mathrm{meV}$ are varied.

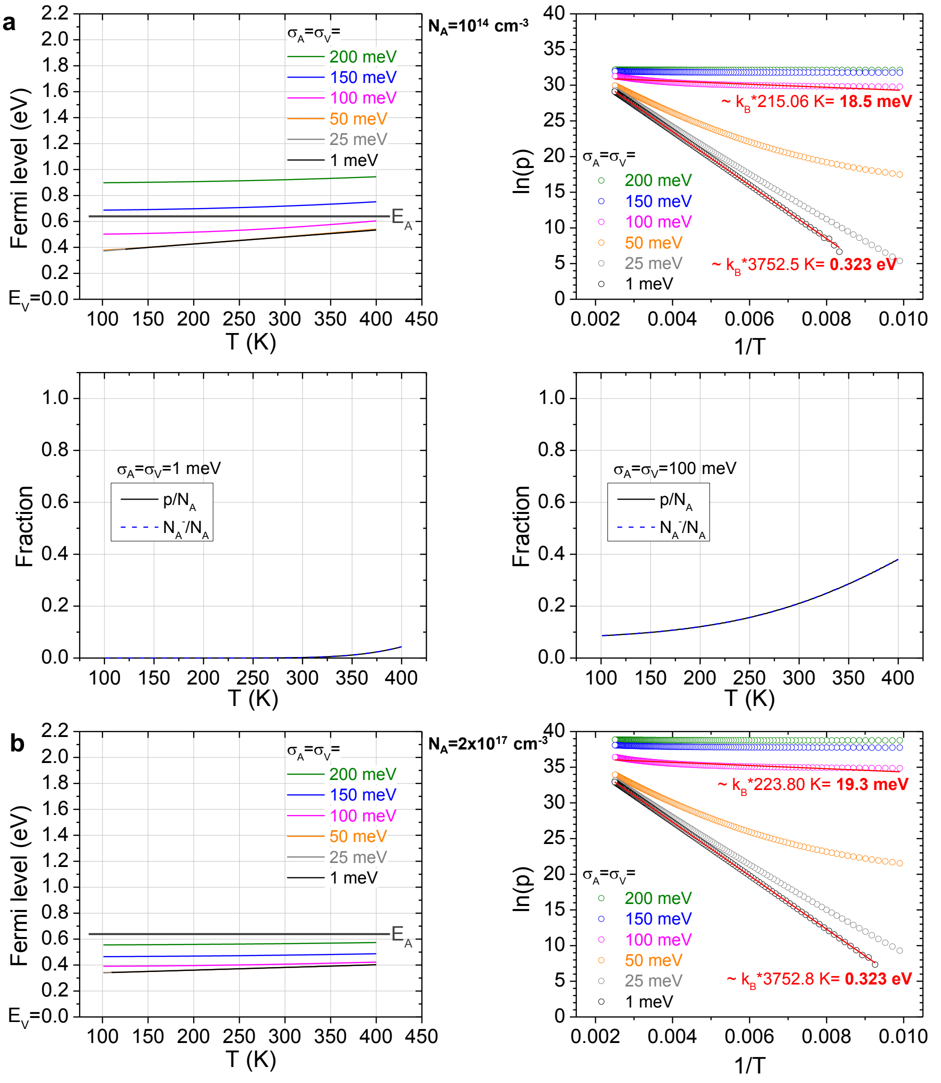

Supplementary Figure 7. Doping model with activation from acceptor states E_A: Thermal activation behavior. Fermi level, free carrier density p and doping efficiency *vs.* temperature T for a two level system consisting of two broadened Gaussians separated by $E_A - E_V = 0.64\,\text{eV}$. The neutrality condition $p = N_A^-$ is solved. The effective valence density is $N_V = 2.4 \times 10^{19}\,\text{cm}^{-3}$. The widths $\sigma_A = \sigma_V = 1 \ldots 150\,\text{meV}$ are varied. The acceptor density is fixed to **(a)** $N_A = 10^{14}\,\text{cm}^{-3}$ and **(b)** $N_A = 2 \times 10^{17}\,\text{cm}^{-3}$.

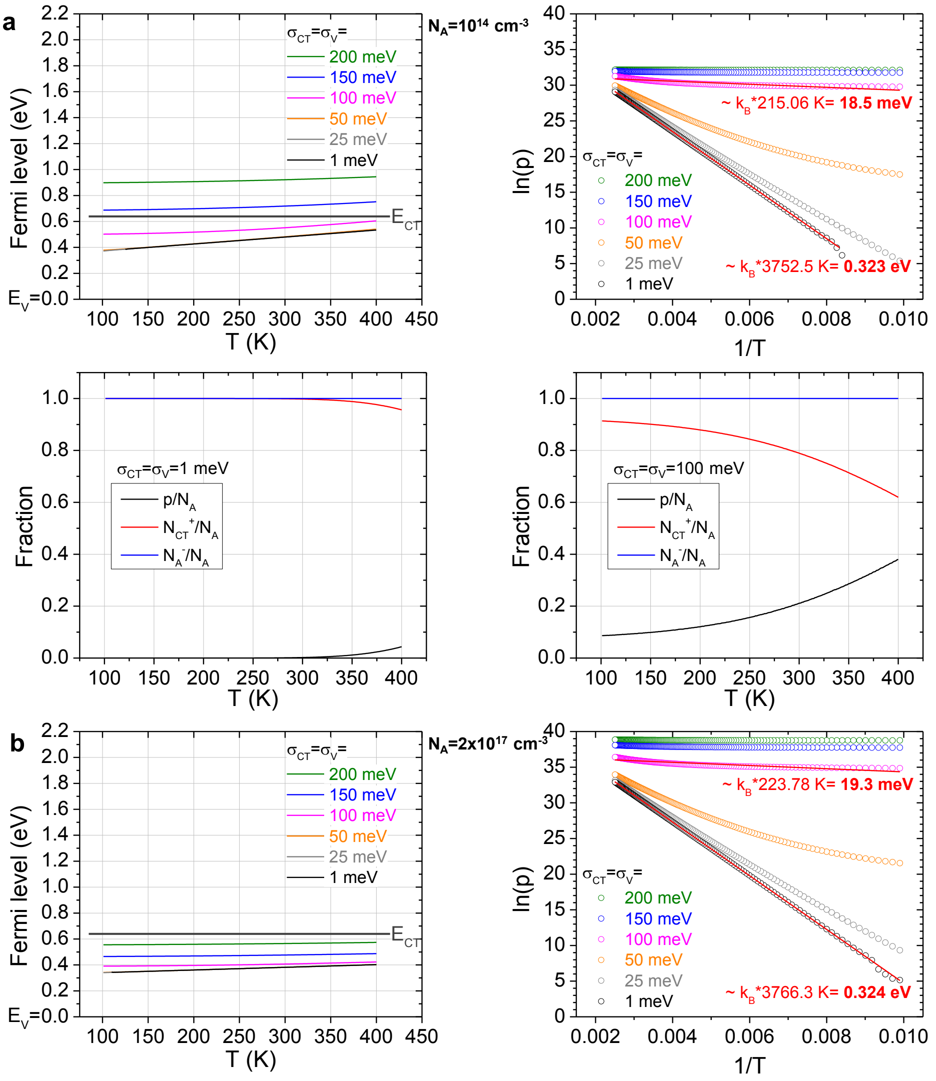

Supplementary Figure 8. Doping model with activation from ICTCs with E^b_{CT}: Thermal activation behavior. Fermi level, free carrier density p and doping efficiency *vs.* temperature T for a two level system consisting of two broadened Gaussians separated by $E^b_{\mathrm{CT}} - E_V = 0.64\,\mathrm{eV}$. The neutrality condition $p + N^+_{\mathrm{CT}} = N^-_A = N_A$ is solved. The effective valence density is $N_V = 2.4 \times 10^{19}\,\mathrm{cm}^{-3}$. The widths $\sigma_A = \sigma_V = 1 \ldots 150\,\mathrm{meV}$ are varied. The acceptor density is fixed to **(a)** $N_A = 10^{14}\,\mathrm{cm}^{-3}$ and **(b)** $N_A = 2 \times 10^{17}\,\mathrm{cm}^{-3}$.

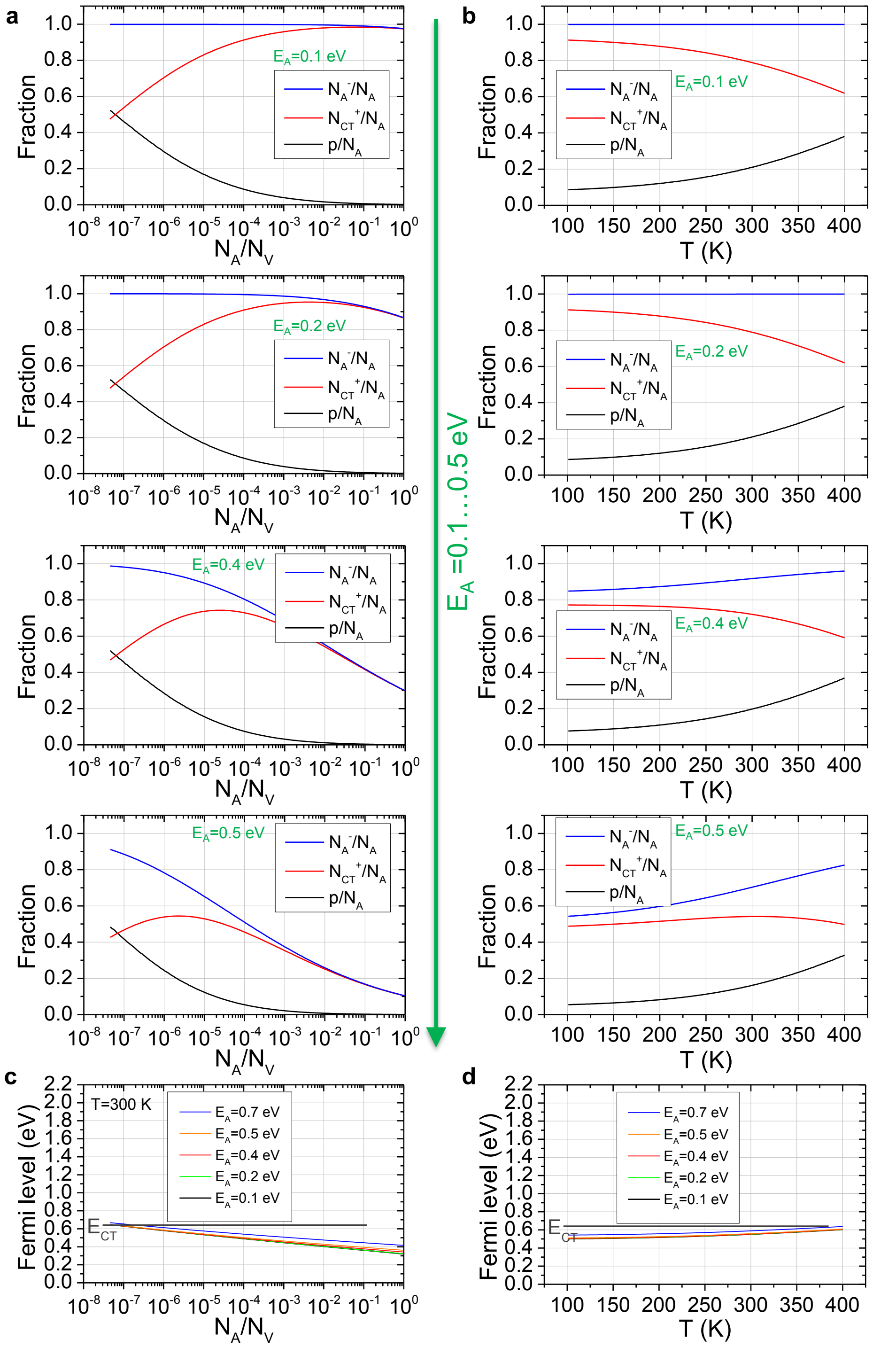

Supplementary Figure 9. Three-level doping model with thermal activation from ICTCs with E^b_{CT} and (deep) acceptor states E_A. Calculated doping efficiency p/N_A and fractions of ionized acceptors N^-_A/N_A as well as occupied ICTCs N^+_{CT}/N_A *vs.* **(a)** the doping concentration or **(b)** temperature T for a three-level system consisting of broadened Gaussians and following the neutrality condition $p + N^+_{\mathrm{CT}} = N^-_A$. Acceptor ionization is given by Supplementary Equation (5). The effective valence density is set to $N_V = 2.4 \times 10^{19}\,\mathrm{cm}^{-3}$, the ICTC binding energy $E^b_{\mathrm{CT}} = 0.64\,\mathrm{eV}$, and the widths $\sigma_A = \sigma_{\mathrm{CT}} = \sigma_V = 100\,\mathrm{meV}$. $T = 300\,\mathrm{K}$ in **(a)** and $N_A = 10^{14}\,\mathrm{cm}^{-3}$ in **(b)**. The acceptor level positions are varied between $E_A = 0.1 \ldots 0.5\,\mathrm{eV}$ to emulate the transition from strong to weak doping. Respective Fermi level positions are plotted in **(c,d)**.

Supplementary Note 2. UPS on ZnPc:F_6-TCNNQ: Depletion layer widths w and doping efficiency at RT

The doping efficiency p/N_A is determined by UPS on incrementally deposited Ag/MeO-TPD(0.7 nm)/ZnPc($d =$ 4.5 nm)/ZnPc:F_6-TCNNQ(x) samples and applying

$$p = N^-_{A,d} = \frac{2\varepsilon_0 \varepsilon_r \Delta V}{e(w^2 + 2wd)} \,. \tag{6}$$

Measured spectra of 4 samples are shown in Supplementary Figure 10 and the estimated depletion layer widths w as well as further parameters are summarized in Supplementary Table 1. The 0.7 nm thin MeO-TPD is applied to de-couple the film growth of ZnPc from the metal substrate and the 4.5 nm ZnPc buffer layer is used to avoid dopant agglomeration at the metal/organic interface, ensuring a homogenous doping profile through the sample.

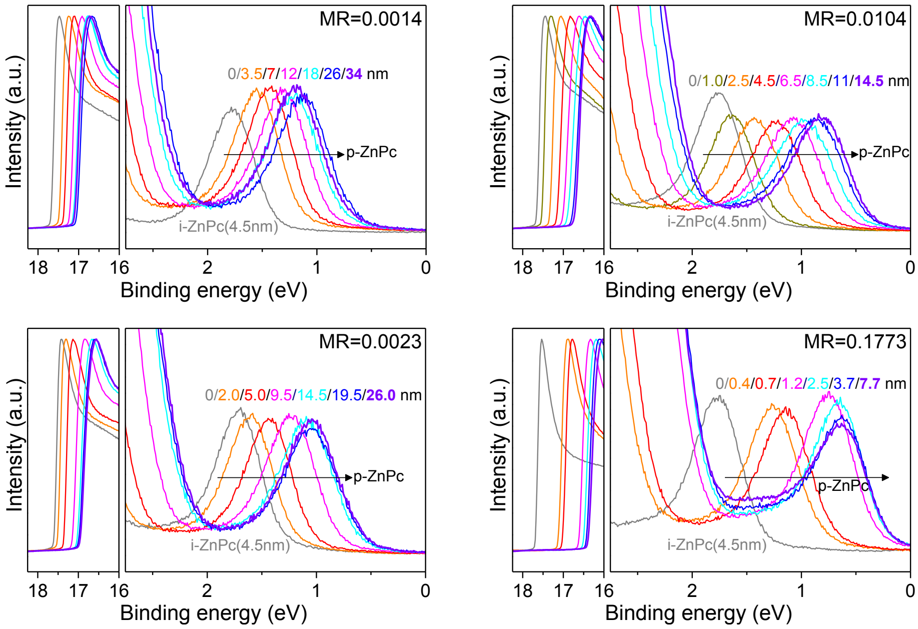

Supplementary Figure 10. UPS on p-doped films at RT. UPS spectra of incrementally deposited Ag/MeO-TPD(0.7 nm)/ZnPc(4.5 nm)/ZnPc:F_6-TCNNQ(x,MR) samples of varying doping ratio.

MR	d (nm)	w (nm)	E_F (eV)	$e\Delta V$ (eV)	p/N_A
0.0014	4.5	23 ± 2.0	0.71	0.62	0.164
0.0023	4.5	17.5 ± 1.2	0.56	1.77	0.197
0.0042	4.5	12.5 ± 1.7	0.46	0.87	0.211
0.0104	4.5	12.0 ± 1.0	0.35	0.89	0.103
0.0279	4.5	9.0 ± 1.5	0.27	1.06	0.064
0.1776	4.5	3.5 ± 0.7	0.20	1.13	0.039

Supplementary Table 1. Depletion layer widths and doping efficiencies in ZnPc:F_6-TCNNQ. Doping efficiency $\eta_{\text{dop}} = p/N_A$ calculated by Supplementary Equation (6) from the depletion layer thickness w in Ag/MeO-TPD(0.7 nm)/ZnPc(4.5 nm)/ZnPc:F_6-TCNNQ(x) samples determined by incremental UPS (cf. Supplementary Figure 10). E_F indicates the Fermi level in the doped layer for $x \geq w$, d the thickness of the intrinsic buffer layer, and $e\Delta V$ the built-in potential. As dielectric constant $\varepsilon_r = 4.0$ is used.[2]

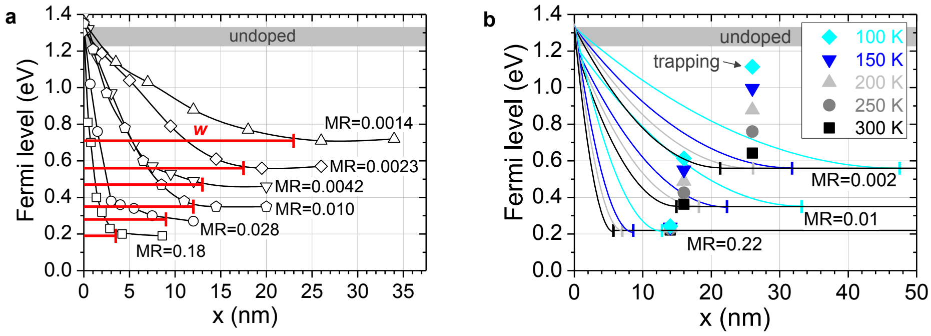

Supplementary Figure 11. Depletion layer widths at metal/organic contacts in UPS. (a) Depletion layer widths w resolved by incremental UPS on Ag/MeO-TPD(0.7 nm)/ZnPc(4.5 nm)/ZnPc:F_6-TCNNQ(x,MR) samples at RT. **(b)** Calculated level bending and depletion widths at Schottky contacts of varying doping ratio and temperature (solid lines, vertical ticks mark the depletion widths $w(T)$) in comparison to UPS on Ag/ZnPc:F_6-TCNNQ(x_m) samples under liquid N_2-cooling (symbols).

Supplementary Note 3. UPS on ZnPc:F_6-TCNNQ: Temperature-dependent depletion width $w(T)$ and level bending

Ag/ZnPc:F_6-TCNNQ(x_m) samples of varying doping ratio are investigated by UPS under temperature variation ($93\,\mathrm{K} < T < 300\,\mathrm{K}$). The evaporated organic layer thicknesses x_m are larger than the expected depletion widths at RT. UPS spectra and determined Fermi level positions are given in Supplementary Figure 12. For samples with $MR \leq 0.01$ a shift of E_F by up to several 100 meV towards mid-gap is found when cooling to 100 K, which is understood to occur due to an increase in the depletion width $w(T)$ beyond the organic layer thickness x_m as illustrated in Supplementary Figure 11(b). For three doping ratios, the $E_F(T)$ UPS values (symbols) are compared to calculated level bendings (lines). The energy level bending within the depletion zone at Ag/ZnPc:F_6-TCNNQ(x) interfaces is given by[3]

$$E_V(x,T) = \Phi_0 - \frac{eN_{\mathrm{CT}}^-}{\varepsilon_0\varepsilon_r}\left(w(T)x - \frac{x^2}{2}\right) \quad \text{for} \quad x \leq w\,, \tag{7}$$

in which the temperature-dependent depletion width

$$w(T) = \sqrt{\frac{2\varepsilon_0\varepsilon_r V_{\mathrm{bi}}(T)}{eN_{\mathrm{CT}}^-}} \tag{8}$$

is defined by the amount of isolated ionized dopants, thus free carrier density, $N_{\mathrm{CT}}^-(T) = p(T)$, and respective built-in potential $V_{\mathrm{bi}}(T) = \Phi_0 - E_F(T)$. The carrier density at diminished temperatures $p(T)$ is estimated from the doping efficiency measured by UPS at RT and the Arrhenius-type deactivation applying $E_{\mathrm{act}} = 20.7\,\mathrm{meV}$. The built-in potential $V_{\mathrm{bi}}(T)$ is determined by the interface potential $\Phi_0 = 1.33\,\mathrm{eV}$ and the (unknown) bulk Fermi level $E_F(T)$ beyond the depletion zone edge. Solutions of the generalized neutrality condition (Supplementary Equation (1)) yield $E_F(T)$ shifts towards the valence states E_V upon cooling. Respective $\mathrm{d}E_F/\mathrm{d}T$ correlations get weakened with increasing energetic disorder, however, do not exceed 100 meV when cooling from RT to 100 K (cf. Supplementary Figure 8). This circumstance is hence expected to only weakly superimpose the depletion layer enlargement effect in the UPS experiments, and therefore, the approximation $V_{\mathrm{bi}}(T) = V_{\mathrm{bi}}(\mathrm{RT})$ is applied in Supplementary Equation (8).
For $MR = 0.22$, the calculated depletion width $w(T = 100\,\mathrm{K}) = 13.8\,\mathrm{nm}$ is yet smaller then the organic layer thickness ($x_m = 14\,\mathrm{nm}$), which is consistent with a practically negligible measured $E_F(T)$ shift. For the lower doped samples, however, the depletion widths clearly increase beyond the organic layer thicknesses. In case of $MR = 0.01$, the measured Fermi level well matches the calculated level bending at 100 K. Furthermore, the calculated depletion width $w(T = 100K) = 33.2\,\mathrm{nm}$ fits well to the value determined by $C(f)$ spectroscopy at 100 K (cf. Fig. 3, main article). The $MR = 0.002$ sample marks the transition to the trap filling regime already at RT, therefore, a much stronger $E_F(T)$ variation is found in UPS than calculated.

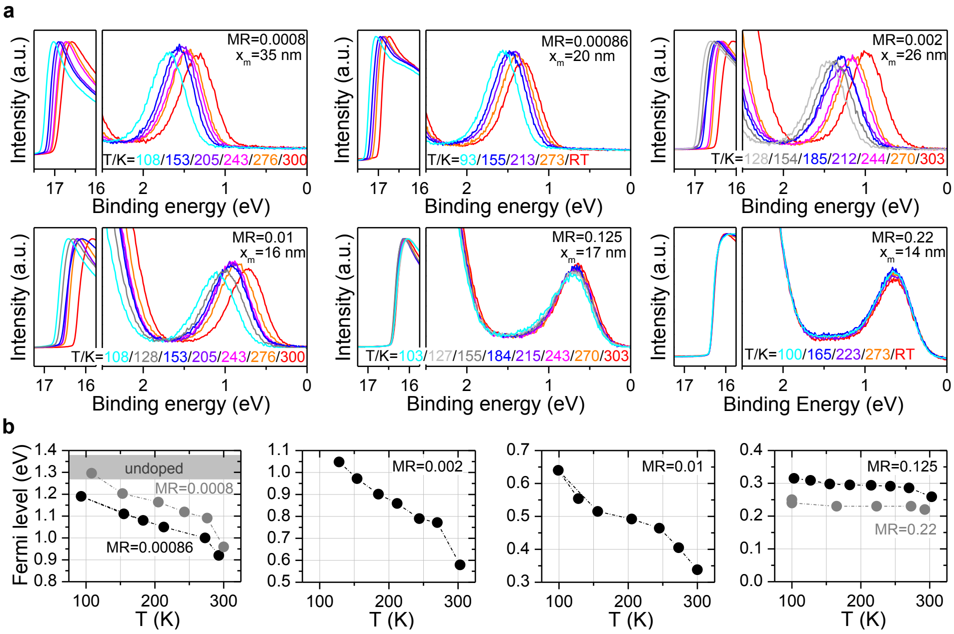

Supplementary Figure 12. UPS on p-doped films under liquid N_2 cooling. (a) UPS spectra and **(b)** determined Fermi level positions w.r.t. the ZnPc HOMO onsets of Ag/ZnPc:F_6-TCNNQ(x_m,MR) samples measured under varying temperature $T = 93\ldots300\,\mathrm{K}$.

Supplementary Note 4. Monte Carlo simulations

Kinetic Monte Carlo simulations, similar to Ref.[4] including randomly chosen dopant sites[5], are performed for a Gaussian disordered energy landscape

$$g(E) = \frac{1}{\sqrt{2\pi\sigma^2}} \exp\left(-\frac{E - E_0}{2\sigma^2}\right) , \tag{9}$$

using nearest neighbor hopping on a simple cubic lattice with periodic boundary conditions. Boxes with $21 \times 21 \times 21$ sites are simulated for dopant concentrations within $2 \times 10^{-4} \ldots 1 \times 10^{-1}$. To check for finite size effects, systems with $51 \times 51 \times 51$ were investigated for concentrations below 1×10^{-2}. Dopant cell energies are randomly distributed following the same standard deviation as for the matrix, with however, a mean value shifted up by 0.05 eV. The on-site interaction energy between dopant cells and charge carriers is limited to the nearest neighbor Coulomb binding energy. This accounts for the high ionization probability that was found in the absorption measurements (cf. main article).[6] Deep traps are included, with a concentration $c_T = 7 \times 10^{-4}$, broadening of 25 meV, and mean depth of $E_T = -1.6\,\mathrm{eV}$.[7] Hopping rates are calculated using Miller-Abrahams rates

$$\nu_{ij} = \nu_0 \begin{cases} \exp\left(-\frac{E_j - E_i}{k_B T}\right) & , E_i < E_j \\ 1 & , E_i \geq E_j \end{cases} , \tag{10}$$

performing at least 10^7 hopping events. Results of 20 different energy landscapes are averaged.

Coulomb interactions of ionized dopants and charge carriers are calculated using Ewald-Summation,[8] with a lattice constant $a = 0.85\,\mathrm{nm}$ and dielectric constant $\varepsilon_r = 4.0$. After each hopping event, the hopping rates of all charge carriers within a radius of 8 cells of the target site are re-calculated.[9] To extract the electrical mobility and to minimize field induced effects, a low external electric field $E_{\mathrm{ext}} = 5 \times 10^6\,\mathrm{V/m}$ is applied.

The electrical conductivity σ_{el} is calculated by multiplying the determined effective mobility μ_{eff} (as described in Ref.[4]) with unit charge e and the *total* number of charge carriers in the simulation, which is equal to the number of dopants $N_A \simeq N_A^-$:

$$\sigma_{\mathrm{el}} = e \times \mu_{\mathrm{eff}} \times N_A \tag{11}$$

E_{ext} (V/m)	ν_0 (s^{-1})	a (nm)	ε	σ_{host} (eV)	$E_{0,host}$ (eV)	σ_{dopant} (eV)	$E_{0,dopant}$ (eV)	σ_T (eV)	E_T (eV)	c_T	T (K)
5e6	1.2e12	0.85	4.0	0.08	0	0.08	0.05	0.025	-1.6	7e-4	300

Supplementary Table 2. Monte Carlo simulation parameters for ZnPc:F_6-TCNNQ. Compilation of the parameters used for simulating the conductivity of ZnPc:F_6-TCNNQ by Monte Carlo. The trap values $\{c_T, E_T, \sigma_T\}$ are taken from Ref.[7].

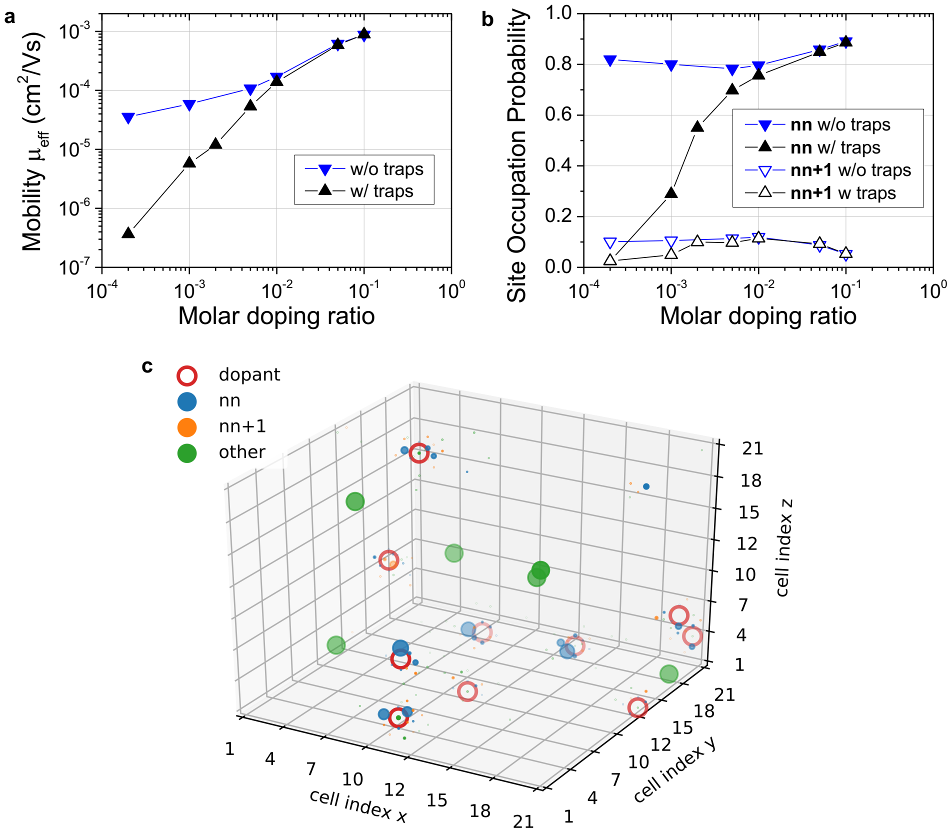

Supplementary Figure 13. Monte Carlo transport simulations on ZnPc:F_6-TCNNQ. **(a)** Effective mobility μ_{eff} determined by Monte Carlo simulations on boxes with 21^3 sites. Filling of deep traps with a relative concentration of $c_T = 7 \times 10^{-4}$, which corresponds to an absolute density of $N_T = 7.2 \times 10^{17}\,\mathrm{cm}^{-3}$ for p-ZnPc,[7] causes a strong rise in μ_{eff} for $MR < 0.01$. **(b)** Corresponding site occupations: Shown is the probability that sites, for which a nearest-neighbor (nn) or next nearest-neighbor (nn+1) site is a dopant, are occupied by a charge carrier (hole). The (nn) probability is much higher than that of (nn+1) sites due to Coulomb attraction of the carriers to ionized dopants. Due to presence of deep traps, the occupation probabilities strongly drop for $MR < 0.005$ each. This is illustrated in **(c)** where the spatial site occupation for holes is shown at $MR = 10^{-3}$. For each site, the plotted dot sizes scales with the respective occupation probability. A large fraction of the holes is found in deep traps states. A respective site occupation plot w/o traps is given in Figure 5 of the main article.
Simulations with 51^3 sites yield same occupation probabilities and mobility trends.

Supplementary References

[1] J. Huang, T. Watanabe, K. Ueno, and Y. Yang. Highly Efficient Red-Emission Polymer Phosphorescent Light-Emitting Diodes Based on Two Novel Tris (1-phenylisoquinolinato-C2, N) iridium (III) Derivatives. *Adv. Mater.* **19**, 739-743 (2007).

[2] M. L. Tietze, W. Tress, S. Pfützner, C. Schünemann, L. Burtone, M. Riede, K. Leo, K. Vandewal, S. Olthof, P. Schulz, and A. Kahn. Correlation of open-circuit voltage and energy levels in zinc-phthalocyanine:C_{60} bulk heterojunction solar cells with varied mixing ratio. *Phys. Rev. B* **88**, 085119 (2013).

[3] S. M. Sze, Physics of Semiconductor Devices. *John Wiley and Sons, New York, 2. edition* (1981).

[4] J. Zhou, Y. C. Zhou, J. M. Zhao, C. Q. Wu, X. M. Ding, and X. Y. Hou. Carrier density dependence of mobility in organic solids: A Monte Carlo simulation. *Phys. Rev. B* **75**, 153201 (2007).

[5] S. Olthof, S. Mehraeen, S. K. Mohapatra, S. Barlow, V. Coropceanu, J.-L. Brdas, S. R. Marder, and A. Kahn. Ultralow Doping in Organic Semiconductors: Evidence of Trap Filling. *Phys. Rev. Lett.* **109**, 176601 (2012).

[6] J. Zhou, Y. C. Zhou, X. D. Gao, C. Q. Wu, X. M. Ding, and X. Y. Hou. Monte Carlo simulation of charge transport in electrically doped organic solids. *J. Phys. D: Appl. Phys.* **42**, 035103 (2009).

[7] M. L. Tietze, P. Pahner, K. Schmidt, B. Lüssem, and K. Leo. Doped Organic Semiconductors: Trap-filling, Impurity Saturation, and Reserve Regimes. *Adv. Funct. Mater.* **25**, 2701 (2015).

[8] S. W. de Leeuw, J. W. Perram, and E. R. Smith, Simulation of electrostatic systems in periodic boundary conditions. I. Lattice sums and dielectric constants. *Proc. R. Soc. Lond. A* **373**, 27-56 (1980).

[9] M. C. Heiber and A. Dhinojwala. Dynamic Monte Carlo modeling of exciton dissociation in organic donor-acceptor solar cells. *J. Chem. Phys.* **137**, 014903 (2012).
